# Meeting of Council

**Published:** 1918-12-07

**Authors:** 


### METROPOLITAN HOSPITAL SUNDAY FUND.

### Meeting of Council.

A MEETING of the Council of the Fund was held at the Mansion House on Friday, November 29, Sir Ernest Tritton, Bart., vice-chairman, in the absence of the Lord Mayor, presiding.

Those present included Lord Cheylesmore, Sir Wm. Church, Bart., Sir Dyce Duckworth, the Archdeacon of Hampstead, Prebendary Ingram, Mr. J. H. Morgan, F.R.C.S., and Prebendary Procter.

The minutes of the previous Council meeting having been approved and signed, the Secretary announced that the following gentlemen had intimated with regret their inability to attend: The Bishop of Islington, the Chief Rabbi, Sir Henry Burdett, K.C.B., Dr. Fleming, the Rev. Dr. Browne, the Rev. Harley Harwood, Mr. Carr Gomme, and others.

The CHAIRMAN moved: "That the annual report of the Council be and is hereby received and adopted, and is ordered to be published." The report showed the record receipts of £92,055, which was some £15,700 more than last year. That, he thought, was admittedly a very fine record for the fourth year of the war. There had been distributed among the various hospitals £85,000, or £15,000 in excess of the amount for last year, and £3,000 had been granted for surgical appliances, on the recommendation of clergy and other ministers. The donations were £5,001 higher than last year, mostly owing to the splendid gift by the American Red Cross Society in recognition of the great work done by our London hospitals for the wounded men belonging to the Allies. As a Council, they valued that gift, not only for its intrinsic worth, but because it was a token of the deep sympathy existing between the British and the United States peoples as well as their Allies, an alliance which, since that gift was offered, had been further cemented on many a blood-stained battle-field, where the troops had fought side by side for liberty, justice, and a righteous cause. The collections had produced over £43,000, which was £5,700 more than last year; and for that they all felt intensely grateful to the clergy and ministers, especially those who had carried the call of the hospitals into the homes of the people by letter, or, still more, by a house-to-house visitation. By such means opportunities to give are afforded to many people, especially the poor, that they would not otherwise have had. This phase of the work was at present only in its infancy, and it showed a prospect of growing into a very lusty youth and manhood. After all, the proportion of people attending places of worship was by no means large, not as large as one would have hoped, but those who stayed away included many generous and ardent souls. He hoped that when this kind of work for the Fund had been developed it would not be found difficult to raise, from among the seven or eight million people in the Metropolis, £100,000. He wished also to say how greatly indebted the Council felt to Messrs. Harrods, Peter Robinson, and Selfridges for having placed part of their valuable advertising spaces at the service of the Fund during the week preceding Hospital Sunday. If still more large advertisers could be induced to do the same, it would help to bring in more money for this great cause. He found it difficult adequately to express what the Fund owed to the press during this year. Never in his recollection since he had been connected with this Fund had the press done more for it than in the year now passed under review. The Committee desired to tender their heartiest thanks for that most valuable service. In conclusion he wished once more to testify to the excellent and indefatigable work which had been done throughout by the Secretary, Mr. Arnold James, and his colleague Mr. Lander. Mr. James was the author of the new movement for placing the Fund's claims before a greatly increased number of people, from which so much was to be expected in the future.

Sir MARCUS SAMUEL said it gave him very sincere pleasure to second the resolution for the adoption of the report. It must be a matter of the deepest gratification to those concerned with the Fund that during the stress and storm and struggle which we had passed through, the hearts of the people had beaten true, and that they had responded more generously than ever to this appeal on behalf of the hospitals, which, as everyone knew, did so much good. This Fund had on its Council men of all creeds, who co-operated in the work of alleviating human suffering. He referred to the great ability and zeal displayed in the work of the Distribution Committee by its head, Sir Wm. Church, who was wholehearted in the cause.

The motion was carried unanimously.

The CHAIRMAN referred, in sympathetic tones, to the death of Mr. Herbert Brooks, one of those who founded the Fund in 1872. Mr. Brooks had been a generous and sympathetic friend of the Fund, and he, the speaker, had the pleasure of his friendship for many years.

SIR DYCE DUCKWORTH proposed that the seventeen members of Council retiring by rotation be recommended for re-election, and that the Rev. E. Marling Roberts, the Rev. W. G. Pennyman, the Rev. Dr. Jowett, and Sir Charles Hanson, Bart., M.P., be recommended for election to fill the vacancies. He was sure the Committee were very sorry to lose the services of the three reverend gentlemen he had referred to, who were enthusiastic workers for the Fund; but he did not doubt that the gentlemen recommended to fill the vacancies would prove to be very zealous. Sir JOHN BELL seconded. The CHAIRMAN pointed out that one of the gentlemen, the Rev. W. G. Pennyman, was the Vicar of St. Mark's, North Audley Street, which sent £2,453, by far the largest gift which had ever come from any church to this Fund. The resolution was unanimously adopted.

It was agreed that the annual meeting of constituents of the Fund should be held at the Mansion House at three o'clock on December 20, and that the next Hospital Sunday should be June 22, 1919.

### THANKS TO THE CHAIRMAN.

Lord CHEYLESMORE moved that a cordial vote of thanks be tendered to Sir Ernest Tritton, not only for his conduct in the chair on this occasion, but for the very great amount of work he had put into the Fund ever since he became its vice-chairman in 1913. During that time the Fund had made great progress, and Sir Ernest could be credited with a large share in it. It had been his Lordship's privilege to be associated with Sir Ernest Tritton on a sub-committee on more than one occasion, and he could testify to the amount of energy he put into whatever he undertook.

The Rev. Prebendary REYNOLDS seconded, and it was carried by acclamation. The CHAIRMAN briefly replied.

